# microRNA-625 inhibits tumorigenicity by suppressing proliferation, migration and invasion in malignant melanoma

**DOI:** 10.18632/oncotarget.14710

**Published:** 2017-01-18

**Authors:** Wei Fang, Yibin Fan, Zhenzong Fa, Jinhua Xu, Hongyu Yu, Pu Li, Julin Gu

**Affiliations:** ^1^ Shanghai Key Laboratory of Molecular Medical Mycology, Department of Dermatology and Venereology, Changzheng Hospital, Second Military Medical University, Shanghai, 200003, China; ^2^ Department of Dermatology, Eastern Hepatobiliary Surgery Hospital, Second Military Medical University, Shanghai, 201805 China; ^3^ Department of Dermatology, Huashan Hospital, Fudan University, Shanghai, 200040, China; ^4^ Department of Pathology, Changzheng Hospital, Second Military Medical University, Shanghai, 200003, China; ^5^ Department of Pediatrics, Ruijin Hospital and Ruijin Hospital North, School of Medicine, Shanghai Jiao Tong University, Shanghai, 200025, People's Republic of China; ^6^ Department of Dermatology, Zhejiang Provincial People's Hospital, Hangzhou, Zhejiang, 310014, China

**Keywords:** malignant melanoma, miR-625, tumorigenesis, SOX2

## Abstract

Dysregulated microRNA (miR)-625 expression has been observed in several kinds of cancer. MicroRNAs are important factors in the development and progression of malignant melanoma, though the clinical significance and function of miR-625 in human malignant melanoma remain unclear. Levels of miR-625 expression were therefore determined in 36 pairs of malignant melanoma and adjacent non-tumor tissue using qPCR. The effects of miR-625 dysregulation on malignant melanoma cell proliferation, wound healing, migration and invasion *in vitro* and tumorigenicity *in vivo* were investigated using CCK-8, transwell assays, and a nude mouse subcutaneous tumor model. Bioinformatics analysis and luciferase reporter system were used to predict and confirm the target gene of miR-625. miR-625 levels were frequently decreased in malignant melanoma. Ectopic expression of miR-625 suppressed proliferation, wound healing, migration, and tumorgenicity in malignant melanoma. Moreover, miR-625 acted, at least in part, by suppressing potential target *SOX2*. These results show that miR-625 is a tumor suppressor that inhibits the development and progression of malignant melanoma, which suggests miR-625 is potentially a new diagnostic marker and therapeutic target of malignant melanoma.

## INTRODUCTION

Malignant melanoma, a skin cancer that arises from malignant transformation of melanocytes, is highly aggressive and metastatic [1]. Patient prognosis is poor and the 5-year survival rate is less than 15% [2, 3]. The malignant melanocytes show aberrant proliferation, resistance to apoptosis, and highly invasive potential and motility capacity, which are important biological characteristics in the aggressive clinical course of the metastatic disease [4, 5]. Researchers have conducted a number of studies of malignant melanoma [6], but the complicated molecular mechanism of malignant melanoma development and progression requires us to make further explorations.

MicroRNAs (miRNAs) are noncoding small RNAs 18 to 22 nucleotides in length that regulate gene expression at the post-transcriptional level, and they influence many biological signaling pathways [7]. miRNAs bind to seed sequences in the 3′-untranslated regions (3′-UTRs) of their targets and induce mRNA degradation or translational suppression [8]. Many studies report significant deregulation of microRNAs expression profiling between tumor cells and cells derived from normal tissue, indicating that miRNAs function as either oncogenes or tumor suppressors in various cancers [9–11].

miR-625 deregulation has been detected in many cancers, including breast cancer [12], colorectal carcinoma [13], hepatocellular carcinoma [14], squamous cell carcinoma [15], and gastric cancer [16]. These studies show that miR-625 is frequently deregulated in tumor tissue or cells, and the decreased expression of miR-625 correlated with the invasiveness and metastasis predict high malignancy and poor prognosis. However, the function of miR-625 in malignant melanoma is largely unknown. A recent study indicates that miR-625 is one of the miRNAs that are downregulated in highly invasive malignant melanoma cells [17].

Therefore, we focused on miR-625 to further investigate its association with malignant melanoma and investigate the function and molecular mechanism of miR-625 in the progression of malignant melanoma and to explore the practicality of using miR-625 as a diagnostic marker and therapeutic target.

## RESULTS

### miR-625 is downregulated in human malignant melanoma

**Figure 1:**
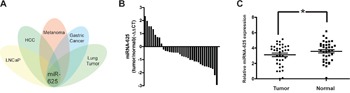
miR-625 is downregulated in malignant melanoma tissues **A**. Overview of cluster analysis of deregulated microRNAs in several common tumors. miR-625 is an overlapping candidate. **B**. miR-625 was detected in melanoma tissues. The data are shown as -^ΔΔ^CT values. **C**. Relative expression of miR-625 in malignant melanoma patients’ tumor tissues compared with normal tissues. Data are shown as mean ± SEM (*P < 0.05).

Through analysis of the related microRNA array data, we found that the abnormal expression of miR-625 is associated with malignancy or the development process in many types of tumors (Figure 1A) [14, 16, 18, 19]], and it was one of downregulated microRNAs in the highly invasive melanoma cell lines [17]. However, the functions and molecular mechanisms of miR-625 have not been investigated in malignant melanoma. To examine the expression levels of miR-625 in malignant melanoma tissue and normal tissue, we performed qRT-PCR analysis on 36 malignant melanoma samples. Results suggested that reduction of miR-625 occurs in approximately 75% of human malignant melanoma (27/36 samples, Figure 1B), and the average relative expression level of miR-625 is significantly downregulated in tumor tissue compared with the non-tumor tissue (Figure 1C, *P < 0.05).

### miR-625 inhibits cell proliferation and clonogenicity of malignant melanoma cells *in vitro*

**Figure 2:**
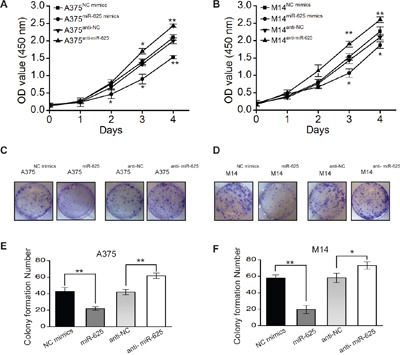
The effect of miR-625 on the proliferation of malignant melanoma cells **A**. Cell proliferation was measured by the WST assay. A375 cells were transfected with miR-625 mimics, NC mimics, anti-625, or anti-NC at a final concentration of 100 nM, at 24 hours after transfection. The WST assay was performed every 24 hours for 4 days. Results are means of three independent experiments ± S.D. (*P < 0.05, **P < 0.01). **B**. M14 cells were transfected with miR-625 mimics, NC mimics, anti-625, or anti-NC at a final concentration of 100 nM, at 24 hours after transfection. The WST assay was performed every 24 hours for 4 days. Results are means of three independent experiments ± S.D. (*P < 0.05, **P < 0.01). **C**. The effect of miR-625 on cell proliferation was evaluated by the plate colony formation assay. A375 cells were transfected with miR-625 mimics, NC mimics, anti-625, or anti-NC, and then seeded onto 6-well plates. The number of colonies was counted on the 14th day after seeding. **D**. M14 cells were transfected with miR-625 mimics, NC mimics, anti-625, or anti-NC, and then seeded onto 6-well plates. The number of colonies was counted on the 14th day after seeding. **E-F**. Colonies containing 50 or more cells were counted. Results are means of three independent experiments ± S.D. (*P < 0.05, **P < 0.01).

Because miR-625 is significantly downregulated in malignant melanoma, it might function as a tumor suppressor. Therefore, we determined whether deregulation of miR-625 in malignant melanoma cells correlates with cell proliferation. To investigate the function of miR-625, mimics or anti-sense oligonucleotides and control oligonucleotides were transfected into malignant melanoma cells A375 and M14, respectively. We examined the effects of miR-625 on cell growth by water-soluble tetrazolium salt (WST) cell-growth assay. The results showed that cell growth was inhibited in the group transfected with miR-625 mimics compared with the cells transfected with control mimics, and the cell growth was promoted by anti-miR-625 compared with the anti-negative control (NC) oligos. (*P < 0.05, **P < 0.01, Figure 2A and 2B).

To further characterize the effect of miR-625 on cell proliferation, a plate colony formation assay was performed. The result showed that the number of colonies from malignant melanoma cells transfected with miR-625 mimics was significantly fewer than the number from the control group, and the number of colonies from cells transfected with anti-miR-625 was higher compared with the control group transfected with anti-NC (Figure 2C and 2D). The number of colonies from A375^miR-625^ and M14^miR-625^ cells were fewer than the number of colonies from control groups A375^NC mimics^ and M14^NC mimics^, and the number of colonies from A375^anti-miR-625^ and M14^anti-miR-625^ cells were higher than the number of colonies from control groups A375^NC mimics^ and M14^NC mimics^ (*P < 0.05, **P < 0.01, Figure 2E and 2F). These findings indicate that miR-625 inhibits proliferation and colony-forming ability of malignant melanoma cells.

### miR-625 inhibits wound-healing ability of malignant melanoma cells

**Figure 3:**
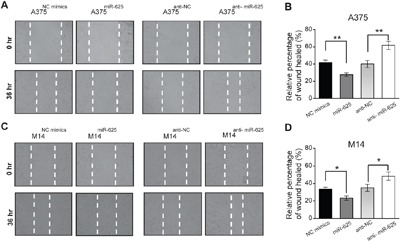
miR-625 inhibits scratch wound-healing ability of malignant melanoma cells **A**. Movement ability of A375^NC mimics^, A375^miR-625^, A375^anti-NC^, or A375^anti-miR-625^ cell lines was detected by scratch wound-healing assays. **B**. Cell migration is quantified as a percentage of wound-healed area. Data represent mean ± SD (**P < 0.01). **C**. Movement ability of M14^NC mimics^, M14^miR-625^, M14^anti-NC^, or M14^anti-miR-625^ cell lines was detected by scratch wound-healing assays. **D**. Cell migration is quantified as percentage of wound-healed area. Data represent mean ± SD (*P < 0.05).

We tested whether miR-625 has an impact on the movement ability of malignant melanoma cells by wound-healing assay. The results are shown in Figure 3A. Restoration of the miR-625 overexpressing cell line A375^miR-625^ slowly closed the scratch wounds compared with the control group 36 hours after scratching. In contrast, the A375^anti-miR-625^ cells were significantly efficient in wound healing (Figure 3B). The results in the M14 cell groups were consistent with the above data (Figure 3C and 3D).

### miR-625 suppresses migration and invasion of malignant melanoma cells *in vitro*

**Figure 4:**
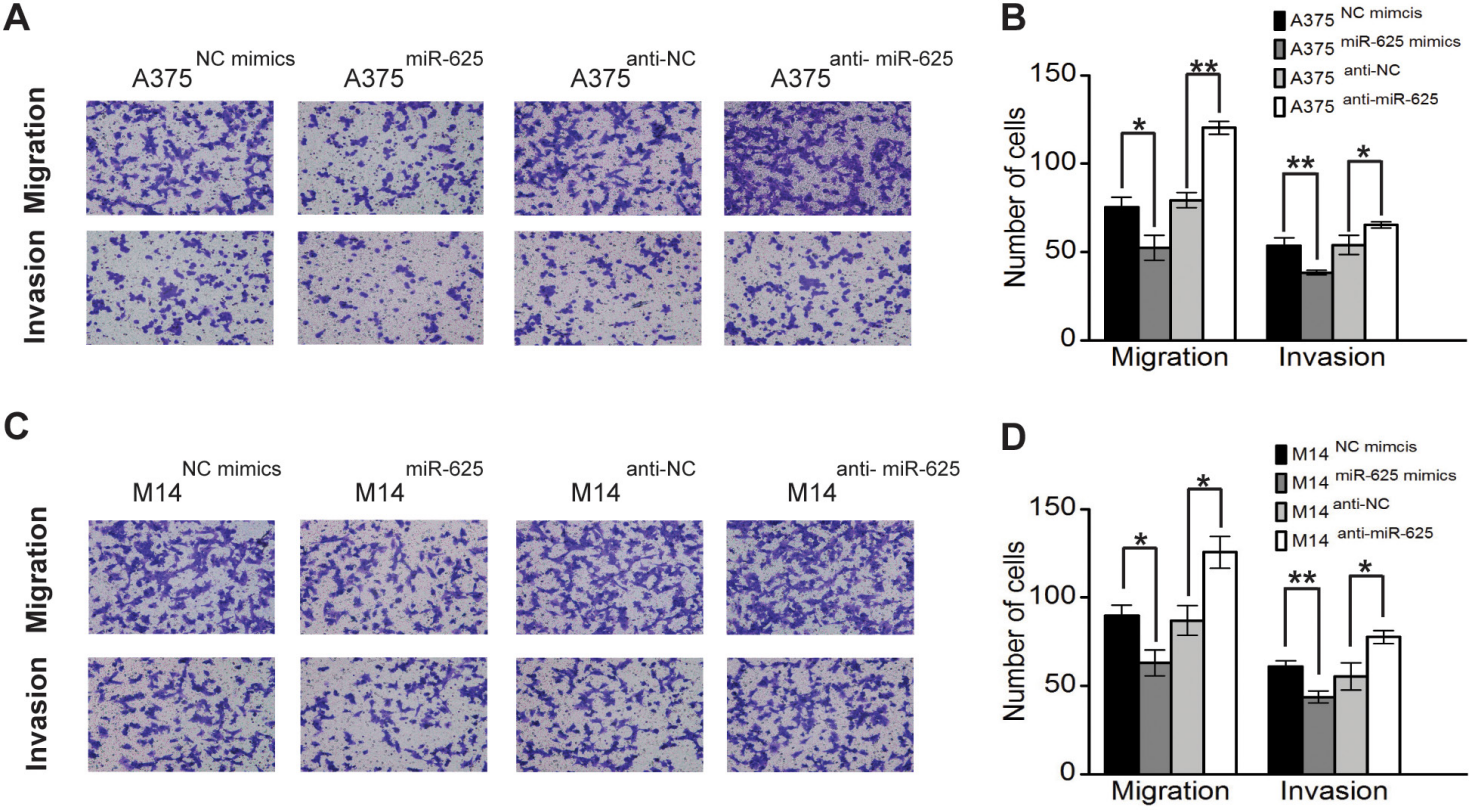
miR-625 inhibits migration and invasion of malignant melanoma cells **A**. Representative photographs of migratory or invasive A375^NC mimics^, A375^miR-625^, A375^anti-NC^, or A375^anti-miR-625^ cells on the membrane (magnification, 100×). **B**. Average number of migratory or invasive A375^NC mimics^, A375^miR-625^, A375^anti-NC^, or A375^anti-miR-625^ cells. (*P < 0.05, **P < 0.01). **C**. Representative photographs of migratory or invasive M14^NC mimics^, M14^miR-625^, M14^anti-NC^, or M14^anti-miR-625^ cells on the membrane (magnification, 100×). **D**. Average number of migratory or invasive M14^NC mimics^, M14^miR-625^, M14^anti-NC^, or M14^anti-miR-625^ cells (*P < 0.05, **P < 0.01). The data represent the mean ±S.D. of three independent experiments.

To further assess the influence of miR-625 on malignant melanoma cells, we explored its effects on cell migration and invasion, a key factor in tumor progression and metastasis. The results showed that ectopic expression of miR-625 significantly suppressed migration and invasion of malignant melanoma cells, whereas knockdown of miR-625 by anti-sense oligos significantly enhanced migration and invasion. As shown in Figure 4, the number of migratory and invasive A375 cells transfected with miR-625 mimics was significantly less than the control group, whereas the number of A375 cells transfected with anti-miR-625 was higher than the control group (**P < 0.01, Figure 4A and 4B), and the results of the M14 cell groups are also consistent. (*P < 0.05, Figure 4C and 4D). Our data show that miR-625 is an important factor in the migration and invasion ability of malignant melanoma cells.

### miR-625 suppresses tumorigenicity of malignant melanoma cells in nude mice *in vivo*

**Figure 5:**
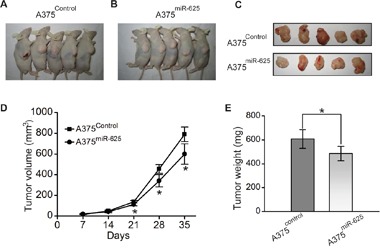
miR-625 inhibits tumorigenicity of malignant melanoma cells *in vivo* **A-C**. Photographs of tumors derived for pSilencer-miR-Control or pSilencer-miR-625 stably transfected A375 cells in nude mice. **D**. Growth kinetics of tumors in nude mice. Tumor diameters were measured every 7 days (*P < 0.05). **E**. Average weight of tumors in nude mice. Means ± S.D. were shown (*P < 0.05).

We next validated whether ectopic expression of miR-625 influences the tumor growth of malignant melanoma cells *in vivo*. To explore the contribution of miR-625 *in vivo*, we carried out mouse xenograft models. Malignant melanoma cells A375 transfected with miR-625 mimics or control mimics were selected and injected subcutaneously into nude mice, and the tumor formation was monitored. As shown in the Figure 5A-5C, the tumors grew progressively in the control group, but were suppressed in the miR-625 ectopic expression group. These mice were euthanized 5 weeks after the injection, and the tumor weights and volume were measured (Figure 5D and 5C). Strikingly, the tumor volumes and weights in the A375^miR-625^ group were significantly less than the volumes and weights in the control group. Thus, these results indicate that miR-625 suppresses tumorigenesis of malignant melanoma cells *in vivo*.

### miR-625 targets the 3′-UTR of *SOX2*

**Figure 6:**
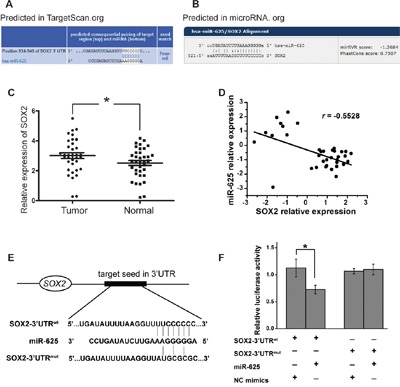
miR-625 targets the 3′-UTR of *SOX2* **A-B**. The putative binding sites of miR-625 in the *SOX2* 3′-UTR region were predicted by miRanda.org and TargetScan.org. The matched seed sequences are indicated by vertical lines. **C**. Relative expression of *SOX2 in malignant melanoma* patients’ tumor tissues compared with normal tissues. Data are shown as mean ± SEM (*P < 0.05). **D**. Linear correlation analysis of *SOX2* and miR-625 expression in melanoma samples. **E**. Schematic graph of the putative binding sites of miR-625 in the *SOX2* 3′UTR and the mutation in miR-625 binding sites. **F**. miR-625 mimics downregulated luciferase activities controlled by wild-type *SOX2* 3′UTR, but did not affect luciferase activity controlled by mutant *SOX2* 3′UTR. The results are means of three independent experiments ± S.D. (*P < 0.05).

To identify how miR-625 functions in malignant melanoma cells, computational prediction of miR-625 targets was performed. We searched putative targets using bioinformatics tools (e.g., TargetScan, Microrna.org, and RNAhybrid) [20–24]. The genes predicted by all the programs were considered candidate targets of miR-625. Among all the hits, sex determining region Y (SRY)-box 2 (*SOX2*) caught our attention (Figure 6A and 6B). We checked the expression levels of *SOX2 in* malignant melanoma tissue and normal tissue. The result showed that the average relative expression level of miR-625 was significantly upregulated in tumor tissue compared with non-tumor tissue (Figure 6C). Moreover, *SOX2* expression levels were inversely correlated with miR-625 levels in tissues (Figure 6D). Then we performed luciferase reporter assays to verify a direct interaction between miR-625 and the 3′UTR of *SOX2*. Luciferase reporters were constructed with either a wild-type *SOX2* 3′UTR sequence that contained the miR-625 binding site (*SOX2*-3′UTR^wt^) or a mutated 3′UTR (*SOX2*-3′UTR^mut^) (Figure 6E). The relative luciferase activity of the *SOX2*-3′UTR^wt^ reporter was apparently suppressed by miR-625 mimics compared with that of *SOX2*-3′UTR^mut^ in an miR-625-dependent manner (Figure 6F). This result strongly indicates that 3′UTR of *SOX2* carries the direct binding seed of miR-625, and miR-625 might target *SOX2* and inhibit its expression.

### *SOX2* can rescue effect of miR-625 in malignant melanoma cells

Since *SOX2* is a potential target of miR-625, it is reasoned that ectopic of *SOX2* could rescue the biological phenotypes caused by miR-625 in malignant melanoma.

**Figure 7:**
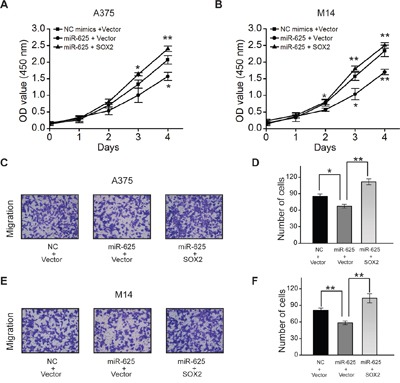
*SOX2* can rescue effect of miR-625 in malignant melanoma **A**. Cell proliferation was measured by the WST assay. A375 cells were transfected with miR-625 mimics, NC mimics, *SOX2*, or Vector control, at 24 hours after transfection. The WST assay was performed every 24 hours for 4 days. Results are means of three independent experiments ± S.D. **B**. M14 cells were transfected with miR-625 mimics, NC mimics, anti-625, or anti-NC at a final concentration of 100 nM and, at 24 hours after transfection. The WST assay was performed every 24 hours for 4 days. Results are means of three independent experiments ± S.D. **C**. Representative photographs of migratory A375^NC mimics+Vector^, A375^miR-625+Vector^, and A375^miR-625+SOX2^ cells on the membrane (magnification, 100×). **D**. Average number of migratory A375 cells. **E**. Representative photographs of migratory M14^NC mimics+Vector^, M14^miR-625+Vector^, and M14^miR-625+SOX2^ cells on the membrane (magnification, 100×). **F**. Average number of migratory M14 cells. The data represent the mean ± S.D. of three independent experiments. **G**. Representative images of spheres as indicated. Scale bar = 50 μm. **H**. Average number of spheres. **I**. Measurement of the size of melanoma spheres. (*P < 0.05, **P < 0.01).

To verify this property, *SOX2* expressing plasmid or control plasmid was introduced into the A375 and M14 cells transiently transfected with miR-625 or control, and then cytological assays were performed to measure cell proliferation and migration. The results showed that ectopic expression of *SOX2* reversed the inhibitory effect of miR-625 on cell proliferation (Figure 7A and 7B) and migration (Figure 7C-7F) in malignant melanoma. Moreover, *SOX2* rescued the effect of miR-625 on sphere formation, which is necessary for cell self-renewal (Figure 7E-7I). Thus, the data provide evidence that inhibition by miR-625 is at least partially related to the function of *SOX2*.

## DISCUSSION

Evidence indicates that miRNAs have very important functions in many biological processes. It can no longer be considered “junk” as it previously had been [25]. In oncology research, miRNAs are shown to promote tumorigenesis and tumor progression.

Although the signatures of miRNAs in malignant melanoma have been well characterized, the functions of deregulated miRNAs in progression and development remain unclear. Deregulation of miR-625 has been reported in various cancers [12–16]. In these studies, the expression levels of miR-625 were significantly lower in tumor tissue or cells compared with corresponding normal tissue or cells, and miR-625 inhibits proliferation, invasion and tumorigenesis *in vitro* or *in vivo*, suggesting that it may serve as a tumor suppressor.

In the present study, we found that the expression of miR-625 decreased significantly in malignant melanoma tissue compared with the normal tissue. The data are consistent with reports in other tumors. Ectopic expression of miR-625 mimics suggested that miR-625 suppresses proliferation, wound healing, migration and invasion in malignant melanoma cells. The nude mouse subcutaneous tumor formation model confirmed that miR-625 inhibits the tumorgenicity in malignant melanoma cells *in vivo*, which is an important aspect of tumor progression.

miRNAs function as oncogenes or tumor suppressors by regulating their targets on the epigenetic level by decreasing translation of target mRNA or increasing degradation of target mRNA [26]. It has been reported that miR-625 targets HMGA1, SCAI, IGF2BP1, and ILK [12–16]. We found that *SOX2* is a potential target of miR-625 in malignant melanoma. *SOX2* is an embryonic stem cell transcription factor. It is an important factor in embryonic development and self-renewal in embryonic stem cells [27], and *SOX2* is one of the determinate transcription factors capable of reprogramming differentiated somatic cells into induced pluripotent stem (iPS) cells [28, 29].

In the field of oncology research, *SOX2* functions as an oncogene in many types of tumors [30–32], including malignant melanoma. *SOX2* inhibits apoptosis via MAP4K4-Survivin signaling in lung cancer cells [33] and promotes metastasis of breast and prostate cancer cells by promoting epithelial-to-mesenchymal transition via the WNT/ β-catenin signal network [34].

**Figure 8:**
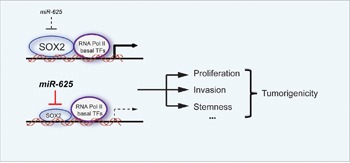
Schematic graph depicting the function of miR-625 in malignant melanoma miR-625 inhibits proliferation, migration, and invasion in melanoma. The down-regulation of miR-625 removes the suppression of its target, *SOX2*, leading to promotion of the tumorigenicity in malignant melanoma.

In previous studies, the expression of *SOX2* was upregulated in melanoma tissues and cells, and *SOX2* invasion, self-renewal, and tumorigenicity of melanoma cells [35, 36]. Our study shows that the expression level of miR-625 is inversely correlated with the expression profile of *SOX2*, and *SOX2* reversed the inhibitory effect of miR-625 in malignant melanoma. The function of tumor suppressor miR-625 is at least partially related to targeting and repressing *SOX2*, (Figure 8).

These findings suggest that miR-625/*SOX2* is an important factor in malignant melanoma tumorigenesis. As a negative regulator of *SOX2*, miR-625 has potential value in precision medical therapy. The combination of protein gene and miRNA has emerged as a tool to predict the efficacy of treatment.

Our data show that miR-625 is frequently downregulated in malignant melanoma. miR-625 suppresses the tumorgenicity and progression of malignant melanoma, suggesting that miR-625 will become a novel diagnostic marker and a new therapeutic target of malignant melanoma.

## MATERIALS AND METHODS

### Ethics statement

This work was approved by the Committee on Ethics of Biomedicine Research, Second Military Medical University. The animal studies were approved by the Committee on Ethics of Biomedicine Research, Second Military Medical University, and carried out in strict accordance with the recommendations in the Regulations for the Administration of Affairs concerning Experimental Animals of the State Science and Technology Commission. The animal model was established under isofluorane anesthesia, and all efforts were made to minimize animal suffering and distress.

### Cell culture

Human malignant melanoma cell lines A375 and M14 were purchased from American Type Culture Collection (ATCC). A375 and M14 were cultured in RPMI-1640 Medium (Corning Cellgro^®^, Manassas, VA, USA) with 10% fetal bovine serum (FBS) (Invitrogen, Carlsbad, CA, USA). Cells were maintained at 37°C with 5% CO_2_ in a humidified incubator.

### RNA isolation and qRT-PCR

Total RNA was extracted from tissues by use of a mirVana™ miRNA Isolation Kit (Applied Biosystems, Foster City, CA, USA) per the manufacturer's instructions. Concentrations and purity of the RNA samples were measured by electrophoresis and spectrophotometric methods. The expression level of miR-625 in tissues was assayed by qRT-PCR and calculated.

### Transient transfection

miR-625 mimics, negative control mimics, miR-625 inhibitors (anti-miR-625) and inhibitor controls (anti-miR-NC) were purchased from GenePharma (Shanghai, Ch ina). Cells were seeded into cell culture plates 20 hours before transfection to ensure 70% cell confluence at the moment of transfection. Transfection of oligonucleotides into malignant melanoma cells was carried out by use of Lipofectamine2000 (Invitrogen, Carlsbad, CA, USA) per the manufacturer's procedure. The oligonucleotides worked at the final concentration of 100 nM.

### Cell proliferation assay

Cell proliferation was assessed by water-soluble tetrazolium salt (WST) assay with the Cell Counting Kit-8 (Dojindo, Kumamoto, Japan) and measured per the manufacturer's instructions. At 24 hours after transfection with mimics or control mimics, malignant melanoma cells were seeded onto 96-well plates (2×10^3^ cells/well), and cell proliferation was documented every 24 hours for 4 days. The number of viable cells was assessed by measurement of the absorbance at 450 nm.

### Wound-healing assay

Wound-healing assay was used to detect the capacity for cell motility. For scratch wound-healing assay, cells were cultured in serum-free medium for 24 hours and wounded with pipette tips. Fresh medium was replaced. The wound-closing procedure was observed for 36 hours and photographs were taken.

### Colony-formation assay

In plate colony-formation assay, malignant melanoma cells were re-suspended in RPMI 1640 containing 10% FBS and layered onto 6-well plates (5×10^2^ cells/well). The cells were incubated for 2 weeks and stained with crystal violet. Colonies containing 50 cells or more were counted.

### Cell migration and invasion assay

Cell migration and invasion assay was performed by use of a transwell chamber (8 μm, 24-well insert; Corning, Lowell, MA, USA). In the migration assay, cells (1×10^5^) in serum-free medium were added to the upper chamber, and medium containing 10% FBS were added to the lower chamber. Cells were then incubated for 24 hours. For the invasion assay, diluted Matrigel (BD Biosciences) was used to coat the membrane of the insert chambers. Cells were cultured for 48 hours under the same conditions. Finally, cells that migrated into or invaded the lower chambers were fixed with methanol, stained with crystal violet, and counted in six random fields.

### *In vivo* tumorigenesis

SPF grade male BALB/c nude mice were purchased from the Institute of Zoology, Chinese Academy of Sciences. Malignant melanoma cells were re-suspended in 0.2 mL of RPMI 1640 and subcutaneously injected into 4-week-old male nude mice (2×10^6^ cells/mouse). The length (L) and width (W) of each tumor were measured every 7 days with calipers, and the volume was calculated by use of the formula (W+L)/2×W×L×0.5236.

### Luciferase reporter activity assay

The 293T cells were cultured in 24-well plates and transfected with 0.2 μg of either wide-type or mutant pMIR/*SOX2* plasmid containing firefly luciferase, together with 0.01 μg of the pRL-TK vector (Promega, Wisconsin, USA) containing renilla luciferase and 1 μg oligonucleotides. Transfection was performed by use of Lipofectamine2000 reagent (Invitrogen). Relative luciferase activity was calculated 36 hours after transfection by the Dual Luciferase Reporter Assay (Promega).

### Statistical analysis

The differences between groups were analyzed by application of the Student *t* test. All statistical analyses were performed by SPSS 15.0 software (SPSS Inc., Chicago, IL, USA), and *P* < 0.05 was considered significant.
